# 2,2′-[Methyl­enebis(sulfanedi­yl)]bis­(pyridine 1-oxide)

**DOI:** 10.1107/S2414314620001716

**Published:** 2020-02-11

**Authors:** David J. Siegel, Alexis N. Howarth, Joseph R. Traver, Patrick C. Hillesheim, Matthias Zeller, Arsalan Mirjafari

**Affiliations:** a Department of Chemistry and Physics, Florida Gulf Coast University, 10501 FGCU Blvd. South, Fort Myers, FL, 33965, USA; b Ave Maria University, Department of Chemistry and Physics, 5050 Ave Maria Blvd, Ave Maria FL, 34142, USA; c Purdue University, Department of Chemistry, 560 Oval Drive, West Lafayette, Indiana, 47907, USA; Howard University, USA

**Keywords:** crystal structure, N-oxide, hydrogen bonding

## Abstract

The title compound, C_11_H_10_N_2_O_2_S_2_, crystallizes with one complete mol­ecule in the asymmetric unit. In the crystal, weak hydrogen bonding is observed between the N-oxide moieties and several C—H units.

## Abstract 2

In the title compound, the two pyridine *n*-oxide moieties are tethered by a bis-thio­ether group.

## Graphical abstract

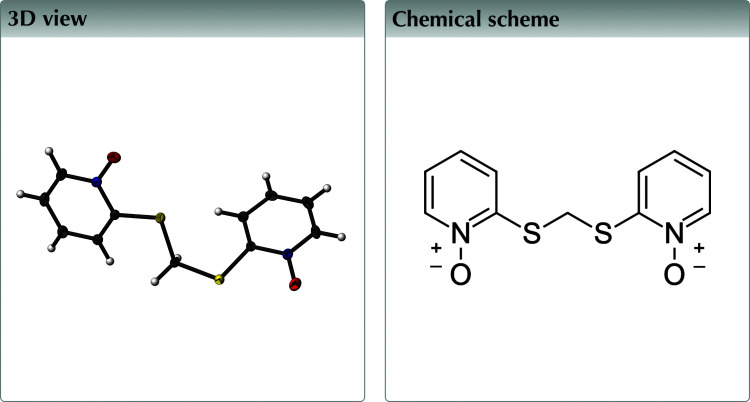

## Structure description

**Figure 1:**
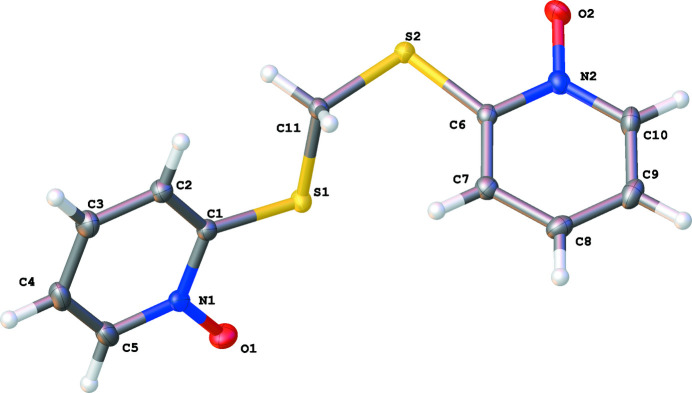
The mol­ecular structure of the title compound shown with 50% probability ellipsoids.

**Figure 2:**
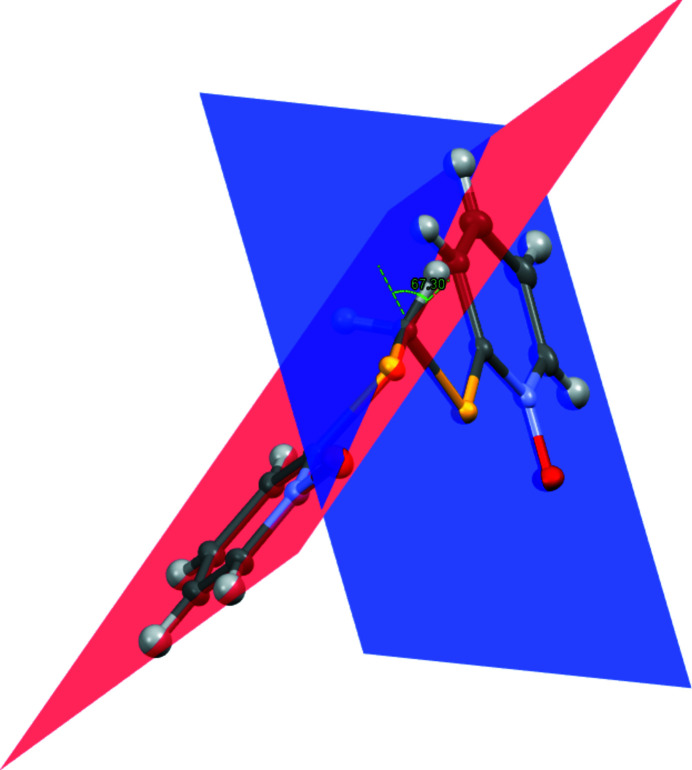
Depiction of the dihedral plane angle of the two pyridine N-oxide moieties visualized within *Mercury* (Macrae *et al.*, 2020 ▸).

The title compound (Fig. 1 ▸) crystallizes in the *P*2_1_ space group with a single mol­ecule in the asymmetric unit. The two nitro­gen–oxygen bonds in the N-oxide moiety exhibit similar lengths [1.307 (3) and 1.309 (3). The two pyridine N-oxide rings exist in a staggered conformation with respect to each other, forming a dihedral angle of 66.55 (9)° (Fig. 2 ▸).

**Figure 3:**
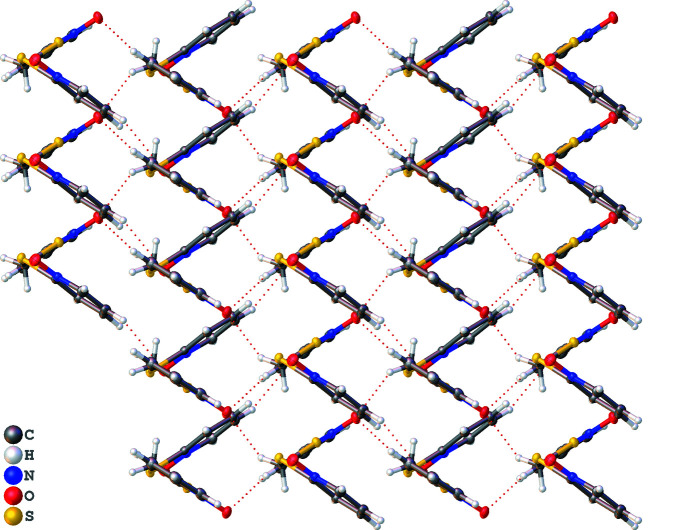
Packing diagram for the title compound as viewed from the [101] direction. Dotted red lines depict the weak hydrogen bonds.

In the extended network, mol­ecules are arranged in a zigzag pattern when viewed along [101] (Fig. 3 ▸); this arrangement facilitates weak hydrogen-bonding inter­actions between adjacent mol­ecules (Table 1 ▸). Both oxygen atoms participate in hydrogen bonding, inter­acting with hydrogen atoms bound to the aromatic rings of the N-oxide moieties. In addition to the inter­actions with aromatic H atoms, O1 is involved in hydrogen bonding with the methyl­ene H atoms from the thio­ether moiety. As a result of the zigzag arrangement of mol­ecules, no π–π stacking is observed.

For related N-oxide crystal structures, see: Rybarczyk-Pirek *et al.* (2018 ▸), Amoedo-Portela *et al.* (2002 ▸), and de Castro *et al.* (2002 ▸)

## Synthesis and crystallization

An oven-dried 100 ml, 24/40 single-necked, round-bottomed flask was charged with a 4 cm oval Teflon-coated stir bar and 2-mercapto­pyridine *N*-oxide sodium salt (1.00 g, 1 equiv.). Dry CH_2_Cl_2_ (4.24 ml, 10 equiv.) was then added to the flask *via* syringe. The flask neck was equipped with a water-jacketed reflux condenser (30.0 cm height, 24/40 joint) with a constant flow of water. The reaction vessel was placed in a pre-heated oil bath and refluxed for an hour under stirring. After the allotted time, the reaction vessel was removed from the oil bath and cooled to room temperature and colorless plates of 2,2′-[methyl­enebis(sulfanedi­yl)]bis­(pyridine-1-oxide) formed over 5 d. The crystals were vacuum filtered and the residual solvent was removed under vacuum (1.40 mm H g) for 12 h to afford 2,2′-[methyl­enebis(sulfanedi­yl)]bis­(pyridine-1-oxide) in high yield (89%).

Crystals suitable for diffraction formed slowly from a CD_2_Cl_2_ solution in an NMR tube.

## Refinement

Crystal data, data collection and structure refinement details are summarized in Table 2 ▸.

## Supplementary Material

Crystal structure: contains datablock(s) I, global. DOI: 10.1107/S2414314620001716/bv4026sup1.cif

Structure factors: contains datablock(s) I. DOI: 10.1107/S2414314620001716/bv4026Isup2.hkl

Supporting information file. DOI: 10.1107/S2414314620001716/bv4026Isup3.cml

CCDC reference: 1982242

Additional supporting information:  crystallographic information; 3D view; checkCIF report

## Figures and Tables

**Table 1:** Hydrogen-bond geometry (Å, °)

*D*—H⋯*A*	*D*—H	H⋯*A*	*D*⋯*A*	*D*—H⋯*A*
C2—H2⋯O1^i^	0.95	2.37	3.292 (3)	165
C9—H9⋯O2^ii^	0.95	2.70	3.405 (4)	131
C11—H11*A*⋯O1^iii^	0.99	2.33	3.159 (3)	141

Symmetry codes: (i) 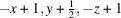 ; (ii) 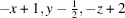 ; (iii) 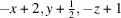 .

**Table 2:** Experimental details

Crystal data	
Chemical formula	C_11_H_10_N_2_O_2_S_2_
*M*_r_	266.33
Crystal system, space group	Monoclinic, *P*2_1_
Temperature (K)	150
*a*, *b*, *c* (Å)	4.1658 (2), 10.4706 (6), 12.7624 (7)
β (°)	95.958 (2)
*V* (Å^3^)	553.67 (5)
*Z*	2
Radiation type	Mo *K*α
μ (mm^−1^)	0.47
Crystal size (mm)	0.55 × 0.16 × 0.04

Data collection	
Diffractometer	Bruker AXS D8 Quest CMOS
Absorption correction	Multi-scan (*SADABS*; Krause *et al.*, 2015 ▸)
*T*_min_, *T*_max_	0.528, 0.747
No. of measured, independent and observed [*I* > 2σ(*I*)] reflections	17033, 4262, 3794
*R*_int_	0.070
(sin θ/λ)_max_ (Å^−1^)	0.772

Refinement	
*R*[*F* ^2^ > 2σ(*F* ^2^)], *wR*(*F* ^2^), *S*	0.041, 0.100, 1.06
No. of reflections	4262
No. of parameters	154
No. of restraints	1
H-atom treatment	H-atom parameters constrained
Δρ_max_, Δρ_min_ (e Å^−3^)	0.56, −0.48
Absolute structure	Flack *x* determined using 1650 quotients [(*I* ^+^)−(*I* ^−^)]/[(*I* ^+^)+(*I* ^−^)] (Parsons *et al.*, 2013 ▸)
Absolute structure parameter	0.05 (4)

Computer programs: *APEX3* and *SAINT* (Bruker, 2018 ▸), *SHELXS97* (Sheldrick, 2008 ▸), *SHELXL2018* (Sheldrick, 2015 ▸, 2018 ▸), *SHELXLE* (Hübschle *et al.*, 2011 ▸), *PLATON* (Spek, 2020 ▸), *OLEX2* (Dolomanov *et al.*, 2009 ▸), *Mercury* (Macrae *et al.*, 2008), *publCIF* (Westrip, 2010 ▸) and *enCIFer* (Allen *et al.*, 2004 ▸).

## full crystallographic data

### Crystal data

**Table:**

C_11_H_10_N_2_O_2_S_2_	*F*(000) = 276
*M**_r_* = 266.33	*D*_x_ = 1.598 Mg m^−^^3^
Monoclinic, *P*2_1_	Mo *K*α radiation, λ = 0.71073 Å
*a* = 4.1658 (2) Å	Cell parameters from 9929 reflections
*b* = 10.4706 (6) Å	θ = 2.5–33.3°
*c* = 12.7624 (7) Å	µ = 0.47 mm^−^^1^
β = 95.958 (2)°	*T* = 150 K
*V* = 553.67 (5) Å^3^	Plate, colourless
*Z* = 2	0.55 × 0.16 × 0.04 mm

### Data collection

**Table:**

Bruker AXS D8 Quest CMOS diffractometer	4262 independent reflections
Radiation source: fine focus sealed tube X-ray source	3794 reflections with *I* > 2σ(*I*)
Triumph curved graphite crystal monochromator	*R*_int_ = 0.070
Detector resolution: 10.4167 pixels mm^-1^	θ_max_ = 33.3°, θ_min_ = 2.5°
ω and phi scans	*h* = −6→5
Absorption correction: multi-scan (SADABS; Krause *et al.*, 2015)	*k* = −16→16
*T*_min_ = 0.528, *T*_max_ = 0.747	*l* = −19→19
17033 measured reflections	

### Refinement

**Table:**

Refinement on *F*^2^	Secondary atom site location: difference Fourier map
Least-squares matrix: full	Hydrogen site location: inferred from neighbouring sites
*R*[*F*^2^ > 2σ(*F*^2^)] = 0.041	H-atom parameters constrained
*wR*(*F*^2^) = 0.100	*w* = 1/[σ^2^(*F*_o_^2^) + (0.0424*P*)^2^ + 0.1658*P*] where *P* = (*F*_o_^2^ + 2*F*_c_^2^)/3
*S* = 1.06	(Δ/σ)_max_ = 0.001
4262 reflections	Δρ_max_ = 0.56 e Å^−^^3^
154 parameters	Δρ_min_ = −0.48 e Å^−^^3^
1 restraint	Absolute structure: Flack *x* determined using 1650 quotients [(*I*^+^)-(*I*^-^)]/[(*I*^+^)+(*I*^-^)] (Parsons *et al.*, 2013)
Primary atom site location: structure-invariant direct methods	Absolute structure parameter: 0.05 (4)

### Special details

**Table:**

Geometry. All esds (except the esd in the dihedral angle between two l.s. planes) are estimated using the full covariance matrix. The cell esds are taken into account individually in the estimation of esds in distances, angles and torsion angles; correlations between esds in cell parameters are only used when they are defined by crystal symmetry. An approximate (isotropic) treatment of cell esds is used for estimating esds involving l.s. planes.
Refinement. C—H bond distances were constrained to 0.95 Å for aromatic C—H moieties. *U*_iso_(H) values were set to 1.2 times *U*_eq_(C).

### Fractional atomic coordinates and isotropic or equivalent isotropic displacement parameters (Å^2^)

**Table:**

	*x*	*y*	*z*	*U*_iso_*/*U*_eq_	
S1	0.81710 (15)	0.68956 (6)	0.58939 (4)	0.01584 (13)	
S2	0.86177 (15)	0.83538 (6)	0.79102 (4)	0.01541 (13)	
O1	0.8980 (5)	0.5371 (2)	0.42557 (17)	0.0230 (4)	
O2	1.0430 (5)	0.7815 (2)	0.99221 (16)	0.0224 (4)	
N1	0.7052 (5)	0.6307 (2)	0.39242 (17)	0.0162 (4)	
N2	0.8365 (5)	0.6943 (2)	0.95562 (16)	0.0162 (4)	
C1	0.6300 (6)	0.7213 (2)	0.46299 (19)	0.0152 (4)	
C2	0.4276 (6)	0.8217 (3)	0.43009 (19)	0.0178 (5)	
H2	0.373352	0.884463	0.479079	0.021*	
C3	0.3049 (7)	0.8300 (3)	0.3252 (2)	0.0221 (5)	
H3	0.167937	0.899069	0.301822	0.026*	
C4	0.3825 (8)	0.7369 (3)	0.2540 (2)	0.0241 (6)	
H4	0.299597	0.741896	0.181928	0.029*	
C5	0.5806 (8)	0.6380 (3)	0.2897 (2)	0.0225 (5)	
H5	0.631713	0.573381	0.241816	0.027*	
C6	0.7073 (6)	0.7022 (3)	0.85309 (18)	0.0139 (4)	
C7	0.4827 (6)	0.6128 (3)	0.8125 (2)	0.0169 (5)	
H7	0.391355	0.618626	0.741250	0.020*	
C8	0.3922 (7)	0.5152 (3)	0.8762 (2)	0.0197 (5)	
H8	0.236511	0.454000	0.849241	0.024*	
C9	0.5303 (7)	0.5072 (3)	0.9797 (2)	0.0223 (5)	
H9	0.471354	0.440113	1.024029	0.027*	
C10	0.7527 (7)	0.5969 (3)	1.0173 (2)	0.0209 (5)	
H10	0.849794	0.590692	1.087870	0.025*	
C11	0.6603 (6)	0.8234 (3)	0.65943 (18)	0.0150 (4)	
H11A	0.692061	0.903548	0.620641	0.018*	
H11B	0.425731	0.811823	0.662873	0.018*	

### Atomic displacement parameters (Å^2^)

**Table:**

	*U*^11^	*U*^22^	*U*^33^	*U*^12^	*U*^13^	*U*^23^
S1	0.0192 (3)	0.0155 (3)	0.0129 (2)	0.0026 (2)	0.00225 (19)	0.0004 (2)
S2	0.0194 (3)	0.0138 (3)	0.0131 (2)	−0.0019 (2)	0.00204 (19)	0.0002 (2)
O1	0.0299 (10)	0.0159 (9)	0.0243 (10)	0.0048 (8)	0.0075 (8)	−0.0012 (7)
O2	0.0259 (10)	0.0239 (10)	0.0165 (9)	−0.0057 (8)	−0.0023 (7)	−0.0028 (7)
N1	0.0205 (10)	0.0149 (10)	0.0141 (9)	−0.0027 (8)	0.0061 (8)	−0.0010 (7)
N2	0.0176 (9)	0.0173 (9)	0.0140 (8)	0.0023 (9)	0.0029 (7)	−0.0005 (8)
C1	0.0188 (11)	0.0143 (11)	0.0129 (9)	−0.0018 (9)	0.0038 (8)	−0.0002 (8)
C2	0.0214 (11)	0.0162 (11)	0.0159 (10)	−0.0005 (9)	0.0030 (8)	−0.0001 (9)
C3	0.0259 (12)	0.0223 (12)	0.0171 (10)	−0.0003 (11)	−0.0020 (9)	0.0022 (11)
C4	0.0302 (14)	0.0280 (14)	0.0135 (11)	−0.0070 (12)	−0.0004 (9)	0.0016 (10)
C5	0.0315 (14)	0.0230 (13)	0.0139 (11)	−0.0070 (11)	0.0060 (10)	−0.0028 (9)
C6	0.0151 (10)	0.0138 (10)	0.0131 (9)	0.0021 (9)	0.0035 (7)	0.0007 (8)
C7	0.0183 (11)	0.0168 (11)	0.0159 (11)	0.0004 (9)	0.0031 (9)	−0.0006 (8)
C8	0.0212 (12)	0.0148 (11)	0.0237 (12)	−0.0018 (10)	0.0053 (9)	0.0003 (9)
C9	0.0259 (13)	0.0190 (12)	0.0235 (13)	0.0030 (10)	0.0093 (10)	0.0070 (10)
C10	0.0248 (13)	0.0229 (12)	0.0154 (11)	0.0031 (11)	0.0044 (9)	0.0063 (9)
C11	0.0186 (11)	0.0148 (11)	0.0119 (9)	0.0012 (9)	0.0022 (8)	−0.0006 (9)

### Geometric parameters (Å, º)

**Table:**

S1—C1	1.749 (3)	C3—H3	0.9500
S1—C11	1.820 (3)	C4—C5	1.373 (4)
S2—C6	1.759 (3)	C4—H4	0.9500
S2—C11	1.802 (2)	C5—H5	0.9500
O1—N1	1.309 (3)	C6—C7	1.385 (4)
O2—N2	1.307 (3)	C7—C8	1.383 (4)
N1—C5	1.361 (3)	C7—H7	0.9500
N1—C1	1.366 (3)	C8—C9	1.388 (4)
N2—C10	1.356 (3)	C8—H8	0.9500
N2—C6	1.365 (3)	C9—C10	1.371 (4)
C1—C2	1.386 (4)	C9—H9	0.9500
C2—C3	1.385 (3)	C10—H10	0.9500
C2—H2	0.9500	C11—H11A	0.9900
C3—C4	1.393 (4)	C11—H11B	0.9900
			
C1—S1—C11	99.12 (11)	C4—C5—H5	119.4
C6—S2—C11	102.00 (12)	N2—C6—C7	120.1 (2)
O1—N1—C5	120.8 (2)	N2—C6—S2	110.72 (19)
O1—N1—C1	118.8 (2)	C7—C6—S2	129.16 (18)
C5—N1—C1	120.4 (2)	C8—C7—C6	119.6 (2)
O2—N2—C10	121.2 (2)	C8—C7—H7	120.2
O2—N2—C6	118.6 (2)	C6—C7—H7	120.2
C10—N2—C6	120.2 (2)	C7—C8—C9	119.5 (3)
N1—C1—C2	120.1 (2)	C7—C8—H8	120.2
N1—C1—S1	111.44 (18)	C9—C8—H8	120.2
C2—C1—S1	128.50 (19)	C10—C9—C8	119.4 (3)
C3—C2—C1	119.5 (2)	C10—C9—H9	120.3
C3—C2—H2	120.3	C8—C9—H9	120.3
C1—C2—H2	120.3	N2—C10—C9	121.1 (2)
C2—C3—C4	120.0 (3)	N2—C10—H10	119.4
C2—C3—H3	120.0	C9—C10—H10	119.4
C4—C3—H3	120.0	S2—C11—S1	110.79 (14)
C5—C4—C3	119.0 (3)	S2—C11—H11A	109.5
C5—C4—H4	120.5	S1—C11—H11A	109.5
C3—C4—H4	120.5	S2—C11—H11B	109.5
N1—C5—C4	121.2 (3)	S1—C11—H11B	109.5
N1—C5—H5	119.4	H11A—C11—H11B	108.1
			
O1—N1—C1—C2	179.7 (2)	C10—N2—C6—C7	1.9 (3)
C5—N1—C1—C2	−0.4 (4)	O2—N2—C6—S2	0.4 (3)
O1—N1—C1—S1	−0.4 (3)	C10—N2—C6—S2	−179.0 (2)
C5—N1—C1—S1	179.46 (19)	C11—S2—C6—N2	179.53 (17)
C11—S1—C1—N1	−179.00 (19)	C11—S2—C6—C7	−1.5 (3)
C11—S1—C1—C2	0.9 (3)	N2—C6—C7—C8	−0.5 (4)
N1—C1—C2—C3	−0.5 (4)	S2—C6—C7—C8	−179.4 (2)
S1—C1—C2—C3	179.6 (2)	C6—C7—C8—C9	−0.7 (4)
C1—C2—C3—C4	0.7 (4)	C7—C8—C9—C10	0.5 (4)
C2—C3—C4—C5	0.1 (4)	O2—N2—C10—C9	178.4 (3)
O1—N1—C5—C4	−179.0 (3)	C6—N2—C10—C9	−2.2 (4)
C1—N1—C5—C4	1.2 (4)	C8—C9—C10—N2	0.9 (4)
C3—C4—C5—N1	−1.0 (4)	C6—S2—C11—S1	−71.09 (14)
O2—N2—C6—C7	−178.7 (2)	C1—S1—C11—S2	−170.31 (14)

### Hydrogen-bond geometry (Å, º)

**Table:**

*D*—H···*A*	*D*—H	H···*A*	*D*···*A*	*D*—H···*A*
C2—H2···O1^i^	0.95	2.37	3.292 (3)	165
C9—H9···O2^ii^	0.95	2.70	3.405 (4)	131
C11—H11*A*···O1^iii^	0.99	2.33	3.159 (3)	141

Symmetry codes: (i) −*x*+1, *y*+1/2, −*z*+1; (ii) −*x*+1, *y*−1/2, −*z*+2; (iii) −*x*+2, *y*+1/2, −*z*+1.
